# Intrabacterial lipid inclusion‐associated proteins: a core machinery conserved from saprophyte *Actinobacteria* to the human pathogen *Mycobacterium tuberculosis*

**DOI:** 10.1002/2211-5463.13721

**Published:** 2023-11-15

**Authors:** Tonia Dargham, Ivy Mallick, Laurent Kremer, Pierre Santucci, Stéphane Canaan

**Affiliations:** ^1^ Aix‐Marseille Univ, CNRS, LISM UMR 7255, IMM FR3479, IM2B France; ^2^ IHU Méditerranée Infection Aix‐Marseille Univ. France; ^3^ Centre National de la Recherche Scientifique UMR 9004, Institut de Recherche en Infectiologie de Montpellier (IRIM) Université de Montpellier France; ^4^ INSERM, Institut de Recherche en Infectiologie de Montpellier France

**Keywords:** bacterial lipid droplets, lipid metabolism, pathogenesis, triacylglycerol

## Abstract

*Mycobacterium tuberculosis* (Mtb), the aetiologic agent of tuberculosis (TB), stores triacylglycerol (TAG) in the form of intrabacterial lipid inclusions (ILI) to survive and chronically persist within its host. These highly energetic molecules represent a major source of carbon to support bacterial persistence and reactivation, thus playing a leading role in TB pathogenesis. However, despite its physiological and clinical relevance, ILI metabolism in Mtb remains poorly understood. Recent discoveries have suggested that several ILI‐associated proteins might be widely conserved across TAG‐producing prokaryotes, but still very little is known regarding the nature and the biological functions of these proteins. Herein, we performed an *in silico* analysis of three independent ILI‐associated proteomes previously reported to computationally define a potential core ILI‐associated proteome, referred to as ILIome. Our investigation revealed the presence of 70 orthologous proteins that were strictly conserved, thereby defining a minimal ILIome core. We further narrowed our analysis to proteins involved in lipid metabolism and discuss here their putative biological functions, along with their molecular interactions and dynamics at the surface of these bacterial organelles. We also highlight the experimental limitations of the original proteomic investigations and of the present bioinformatic analysis, while describing new technological approaches and presenting biological perspectives in the field. The *in silico* investigation presented here aims at providing useful datasets that could constitute a scientific resource of broad interest for the mycobacterial community, with the ultimate goal of enlightening ILI metabolism in prokaryotes with a special emphasis on Mtb pathogenesis.

## Graphical abstract

Dargham *et al*. perform an *in silico* analysis of three independent intrabacterial lipid inclusions (ILI)‐associated proteomes to identify orthologous proteins in *Mycobacterium tuberculosis*. Using this approach, they identify 168 candidate proteins among which 70 are strictly conserved. By performing additional analysis, they investigate and discuss the functional and structural binding features of these proteins to further understand their contribution to ILI metabolism.

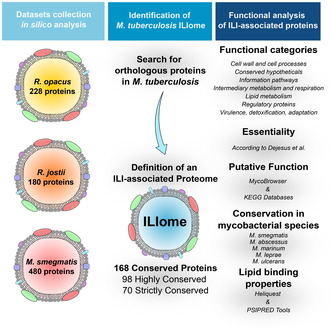

**Abbreviations**
AG: Arabinogalactan
BLASTp: Basic Local Alignment Search Tool program
BTZ: Benzothiazinones
Dgat/Tgs: Diacylglycerol acyltransferase/triacylglycerol synthases
DNB: Dinitrobenzamide
Dos: Dormancy survival regulon
FASII: Fatty acid synthase II
FC: Functional category
FFA: Free fatty acids
HC: Highly conserved
ILI: Intrabacterial lipid inclusions
KEGG: Kyoto Encyclopedia of Genes and Genomes
LAM: Lipoarabinomannan
LB: Lipid body
LD: Lipid droplet
LI: Lipid inclusion
Mabs: *Mycobacterium abscessus*
Mlep: *Mycobacterium leprae*
Mmar: *Mycobacterium marinum*
Msmeg: *Mycobacterium smegmatis*
Mtb: *Mycobacterium tuberculosis*
Mul: *Mycobacterium ulcerans*
PLIN: Perilipin
SC: Strictly conserved
TAG: Triacylglycerol
TB: Tuberculosis

Lipid droplets (LD), also referred to as lipid bodies (LBs) or lipid inclusions (LIs), are lipid‐rich organelles synthesized by numerous eukaryote organisms, including plants and mammals [1, 2, 3]. These structures are also synthesized by prokaryotes, as intrabacterial lipid inclusions (ILI) [4, 5, 6, 7], underscoring the widely conserved nature of these organelles among multiple kingdoms [1, 2, 3]. Lipid droplet and ILI are essentially composed of neutral lipids surrounded by an amphipathic monolayer of phospholipids with proteins that are dynamically interacting at their surface [6, 8]. In addition to a common compositional architecture, they often share conserved proteins with multiple biological functions that are essential for their biogenesis, maintenance/integrity, and degradation [9].

Among prokaryotes, species that belong to the genus *Nocardia*, *Dietzia*, *Gordonia*, *Streptomyces*, *Rhodococcus*, and *Mycobacterium* have been reported to produce high amounts of triacylglycerol (TAG) and detectable ILI structures upon specific *in vitro* culture conditions, suggesting that all can synthesize TAG‐containing ILI [4, 7]. In this context, some *Rhodococcus* and *Mycobacterium* species have been widely used as model systems during the last decades to delineate the processes of ILI formation, maintenance, and consumption at both cellular and molecular levels [10, 11, 12, 13, 14, 15, 16]. Intrabacterial lipid inclusion biosynthesis was proposed to be initiated in specific and spatially distinct microdomains located at the inner leaflet of the plasma membrane [17]. In these globular microstructures, TAG keeps accumulating under the co‐action of multiple enzymes, notably the diacylglycerol acyltransferase/triacylglycerol synthases (Dgat/Tgs) [17]. With the increasing level of TAG, these globules further expand to form a premature ILI that is surrounded by a phospholipid monolayer, which is later released freely in the cytosol to form a mature organelle [17]. This model, proposed almost 20 years ago, is still to date the reference biological model for ILI biosynthesis in prokaryotes [17]. Finally, upon carbon starvation or nutrient‐rich favorable culture conditions, ILI are hydrolyzed by lipolytic enzymes to provide free fatty acids (FFA) and acetyl‐CoA used as a major energy source for cellular homeostasis, maintenance, and regrowth, substantiating the importance of lipid metabolism in bacterial physiology [14, 16, 18].

In the context of tuberculosis (TB), independent studies reported that host‐derived lipids and ILI metabolism are keys in the tubercle bacilli pathogenicity [19, 20, 21, 22, 23, 24]. Indeed, *Mycobacterium tuberculosis* (Mtb) mutants that are unable to synthesize or hydrolyze TAG‐containing ILI display important fitness defects and impaired survival *in vitro*, *in cellulo*, and in *in vivo* biological systems that recapitulate TB persistence and reactivation stages [23, 25]. Moreover, the presence of ILI‐positive Mtb in sputum samples from patients with active TB reinforces the idea these organelles may be essential for the Mtb physiopathological lifestyle with important clinical implications [13, 26, 27, 28].

To date, while the sequential anabolic and catabolic mechanisms governing ILI metabolism start to be well‐documented, the nature and function of ILI‐associated proteins remain elusive. Recent investigations combining biochemical and proteomic approaches unraveled a subset of potential proteins associated with ILI in *Rhodococcus* and *Mycobacterium* species [29, 30, 31, 32]. However, none of these studies have been directly performed with Mtb. Thus, the composition of the Mtb ILI‐associated proteome remains unknown. Since ILI have been described in both pathogenic and nonpathogenic mycobacteria, including *M. smegmatis* (Msmeg) [13, 14, 15, 32], *M. abscessus* (Mabs) [14, 33], *M. avium* (Mav) [34], *M. marinum* (Mmar) [35], *M. ulcerans* (Mul) [36], *M. leprae* (Mlep) [37], and members of the *M. tuberculosis* complex [26, 31, 38, 39, 40], we speculated that proteins/enzymes involved in ILI metabolism identified in *Actinobacteria* may also be conserved in Mtb.

In this study, we capitalized from previously published proteomic investigations in *Rhodococcus* and *Mycobacterium* to identify putative ILI‐associated orthologous proteins in Mtb, and computationally define and characterize a core ILI‐associated proteome in the tubercle bacilli. In particular, we focus our analysis on proteins known or described as key actors in lipid metabolism and explore their putative function in ILI metabolism and mycobacterial physiology. We also discuss the plausible binding features of proteins associated with the surface of ILI. This article aims at providing new visions and insights into the biology of mycobacterial ILI to address relevant challenges and provide new scientific directions that could benefit the scientific community.

## Methods

### Computational prediction and conservation of the Mtb ILI‐associated proteome

The ILI‐associated proteomes from *Rhodococcus jostii* RHA1 (Rjos—taxid:101510), *Rhodococcus opacus* PD630 (Rop—taxid:543736) and *Mycobacterium smegmatis* mc^2^155 (Msmeg—taxid:246196) were obtained from three independent studies [29, 30, 32]. Regarding the Rjos and Rop datasets, only the ILI‐associated proteins found in two independent shotgun proteomic experiments were included in our analysis [29, 30]. Following this specific criterion, 228 and 180 individual proteins were listed in tables to constitute the Rjos and Rop ILI‐associated proteomes, respectively. Regarding the Msmeg dataset, we kept the selection criteria set up by Armstrong and colleagues [32] and further included 480 individual proteins in our analysis to constitute the Msmeg ILI‐associated proteome. In both cases, each individual protein sequences were analyzed by using the Basic Local Alignment Search Tool program BLASTp (https://blast.ncbi.nlm.nih.gov/Blast.cgi) [41] to retrieve putative orthologs in the *M. tuberculosis* H37Rv proteome (Mtb—taxid:83332). Scoring parameters were set as default parameters using a BLOSUM62 matrix with gap existence costs of 11 and gap extension costs of 1. The conditional compositional score matrix adjustment option was used. The maximum alignment score was used to select the best hits and identified Mtb proteins. For each putative ortholog identified, we complemented our research using the Kyoto Encyclopedia of Genes and Genomes (KEGG) database (https://www.genome.jp/kegg/) and the MycoBrowser database (https://mycobrowser.epfl.ch/) [42, 43]. We collected information regarding (a) the gene/protein id (H37Rv gene/protein number), (b) the protein sequence, (c) the corresponding annotated functional category (FC), and finally (d) the *in vitro* gene essentiality as assessed by saturating transposon mutagenesis, according to Dejesus and colleagues [44]. Then, the *in silico* Mtb ILI‐associated core proteome was defined by listing putative orthologs that are conserved in multiple datasets. Finally, this putative core proteome was used to identify conserved mycobacterial ILI‐associated proteins in the following species, *M. smegmatis* mc^2^155 (Msmeg—taxid:246196), *M. abscessus* ATCC 19977 (Mabs—taxid:561007), *M. marinum* M (Mmar—taxid:216594), *M. leprae* TN (Mlep—taxid:272631), and *M. ulcerans* Agy99 (Mul—taxid:362242).

### Structural and putative binding properties of Mtb putative ILI‐associated proteins

Structural and binding properties of the conserved protein candidates across multiple datasets were analyzed by combining bioinformatic softwares. First, the primary sequence of each protein was subjected to the PSI‐blast‐based secondary structure PREDiction program v4.02 (PSIPred) (http://bioinf.cs.ucl.ac.uk/psipred/) [45, 46] to predict the alpha helices. To screen and further identify putative ILI‐targeting alpha helices, the Heliquest software (http://heliquest.ipmc.cnrs.fr) [47] was used by applying the parameters reported by Armstrong and colleagues [32]. Briefly, the hydrophobicity parameter ‘H’ ranged from 0.40 to 0.60, the mean hydrophobic moment ‘μH’ ranged from 0.40 to 0.75, and the net charge ‘z’ was set between − 4 and +4. The amino acid composition was set to 2 polar residues, 1 uncharged residue (Ser, Thr, Asn, Gln, and His), no glycine, 10 charged residues, no proline at i, i + 3/*n* − 3 n, and no cysteine. No geometric rules and no BlackList filters were applied. From the putative amphipathic helices identified using this screening procedure, the most interesting candidates were displayed as an helical wheel diagram. Finally, potential hydrophobic binding regions or potential electrostatic interactions were assessed manually using the experimentally determined 3D structures of the candidate proteins available from the Protein Data Bank (https://www.rcsb.org) or, alternatively, by using models generated from the AlphaFold v2.0 prediction software (https://alphafold.ebi.ac.uk/) [48] and visualized using the ChimeraX software and its in‐built tools (https://www.cgl.ucsf.edu/chimerax/) [49].

## Results and Discussion

### Identification of Mtb orthologous ILI‐associated proteins from Rjos, Rop, and Msmeg proteomic datasets

**Fig. 1:**
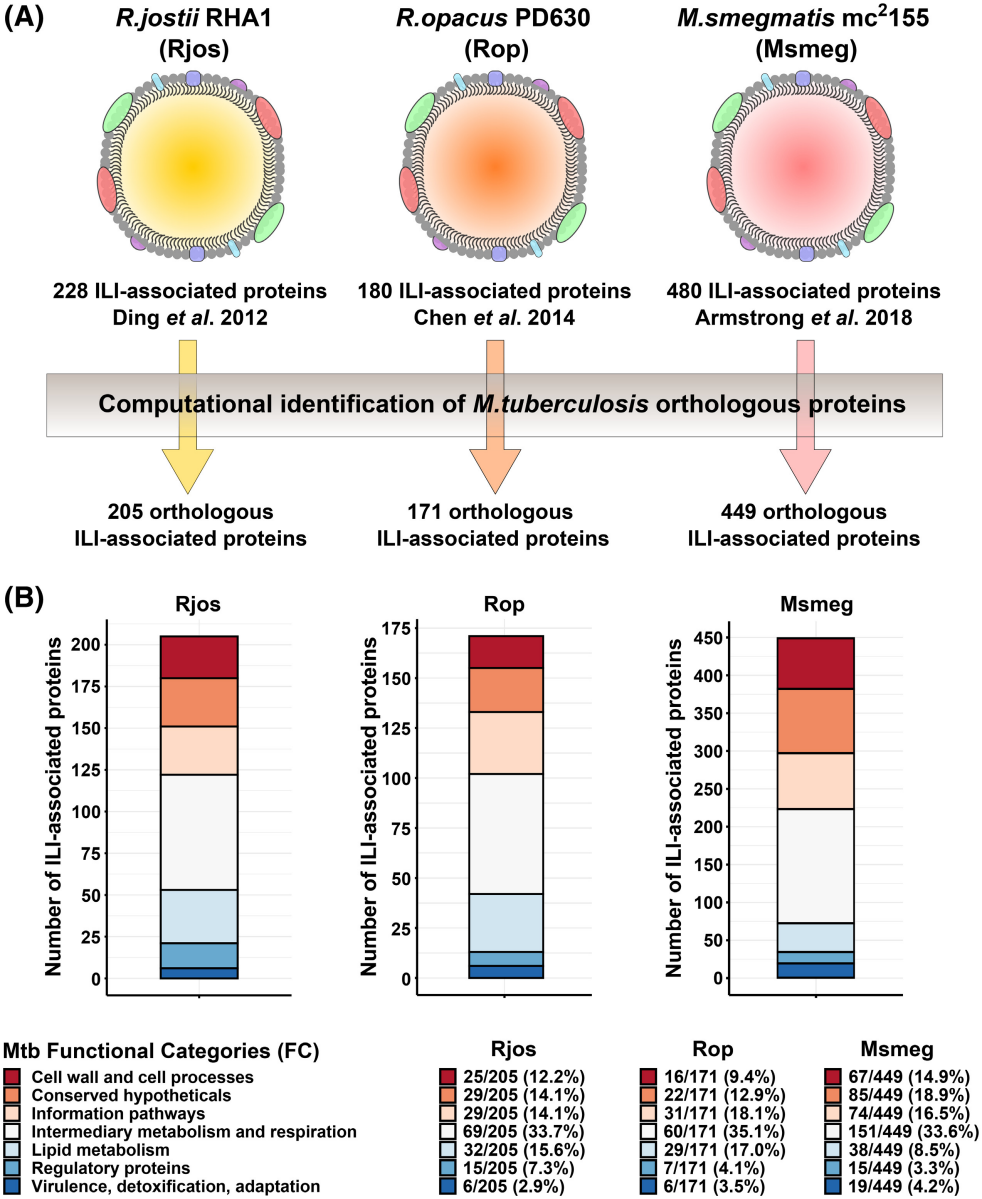
Computational identification of Mtb orthologous ILI‐associated proteins. (A) Schematic representation of the experimental workflow performed in this study. Previously identified ILI‐associated proteins from three independent studies in Rjos, Rop, and Msmeg were selected, and their orthologous proteins in Mtb were identified by using the Basic Local Alignment Search Tool program BLASTp, KEGG, and the MycoBrowser programs. (B) Distribution of the identified ILI‐associated protein orthologs from each organism based on their respective FC. FCs of each protein were obtained based on Mtb original genome annotation and include proteins of ‘*Cell wall and cell processes*’, ‘*Conserved hypothetical*’, ‘*Information pathways*’, ‘*Intermediary metabolism and respiration*’, ‘*Lipid metabolism*’, and ‘*Regulation and virulence detoxification, adaptation*’.

To identify mycobacterial proteins that may be commonly associated with ILI, an *in silico* analysis was initiated based on previously characterized ILI‐associated proteomes. We capitalized from three available datasets established by performing proteomic analysis of ILI‐enriched fractions obtained after sucrose gradient isolation [50]. *R jostii* RHA1 (Rjos), *R. opacus* PD630 (Rop), and *M. smegmatis* mc^2^155 (Msmeg) original datasets [29, 30, 32], consisting of 228, 180, and 480 individual proteins forming the basis of their corresponding ILI‐associated proteomes, respectively (Fig. 1A). Within each of these datasets, we searched for potential orthologous proteins in Mtb. BLASTp analyses identified putative candidates in the Mtb H37Rv proteome (NCBI taxid:83332). We complemented our analysis using the KEGG and the MycoBrowser database [42, 43]. Our analysis unraveled 205/228 (90%), 171/180 (95%), and 449/480 (93.5%) orthologs in Mtb as compared to the previously identified ILI‐associated proteins in Rjos, Rop, and Msmeg, respectively (Fig. 1A). Specific information of each of these putative targets are available in Tables [Link], [Link].

Since Mtb, is by far the most studied bacteria belonging to the *Actinobacteria* phylum, we took advantage of its well‐documented genome and proteome to further characterize the identified orthologous proteins [51, 52, 53]. According to Cole and colleagues' initial annotation, latter updated by Camus *et al*., the Mtb genome and its corresponding coding sequences were classified into 11 FCs based on bioinformatic comparison [51, 52, 53]. From this, the specific Mtb FC was assigned for each orthologous protein (Fig. 1B). Only nine FCs were potentially of interest since ‘*Stable RNAs*’ and ‘*Insertion sequences and phages*’ FC are not applicable to proteins. In our analysis, only seven out of the nine FC were represented in the Rjos, Rop, and Msmeg datasets as no orthologous proteins belonging to ‘*Unknown*’ and ‘*PE/PPE*’ FC were identified. Overall, the partitioning of the 205, 171, and 449 identified orthologous proteins were similar in the seven FC (Fig. 1B). Surprisingly, proteins classified into the ‘*Intermediary metabolism and respiration*’ FC represented approximately one‐third of each dataset with 69/205 (33.7%), 60/171 (35.1%), and 151/449 (33.6%), respectively (Fig. 1B). On the contrary, only 32/205 (15.6%), 29/171 (17.0%), and 38/449 (8.5%) of the proteins identified belong to the ‘*Lipid metabolism*’ FC, suggesting that proteins involved directly into lipid anabolism, catabolism, or transport are not overrepresented at the surface of these organelles. We next collected information about the essentiality of each candidate in Mtb based on the seminal work of Dejesus and colleagues [44]. Results are available for each dataset in Tables [Link], [Link] and show that 68/205 (33.2%), 69/171 (40.4%), and 140/449 (31.2%) of the putative ILI‐associated protein‐encoding genes are essential for Mtb *in vitro*. This suggests that ILI shelter proteins that display critical physiological functions for cellular homeostasis and growth.

### Identification of 168 ILI‐associated proteins that are conserved across multiple proteomic datasets—Definition of a ‘minimal’ ILIome core

**Fig. 2:**
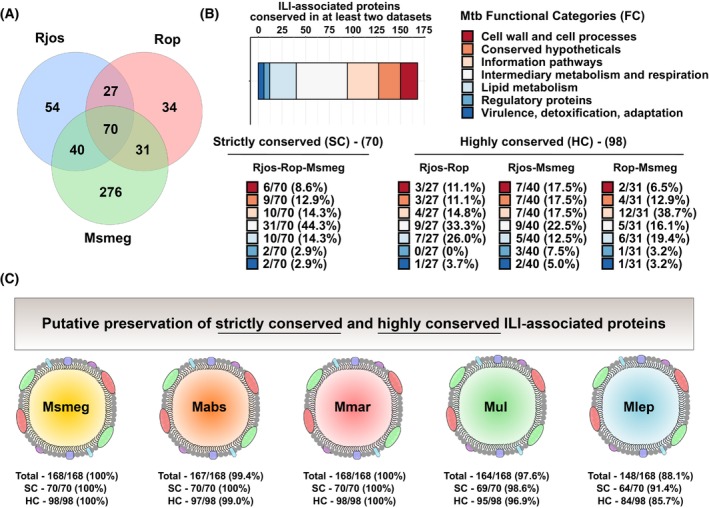
Analysis of ILI‐associated protein conservation across the multiple proteomic datasets. (A) Venn diagram representation of Mtb orthologous proteins repartition from the three datasets analyzed, and identification of proteins that are conserved across the three species. (B) Repartition of the 168 identified ILI‐associated protein orthologs that are contained in at least 2 datasets based on their respective FC (Top panel). Repartition of the identified ILI‐associated protein orthologs that are SC across Rjos, Rop, and Msmeg datasets or HC across two datasets (Bottom panels). FCs of each protein were obtained based on Mtb original genome annotation and includes proteins of ‘*Cell wall and cell processes*’, ‘*Conserved hypothetical*’, ‘*Information pathways*’, ‘*Intermediary metabolism and respiration*’, ‘*Lipid metabolism*’, and ‘*Regulation and virulence detoxification, adaptation*’. (C) Schematic representation of the conservation levels of the 168 ILI‐associated proteins identified in Mtb within five other mycobacterial species including Msmeg, Mabs, Mmar, Mul, and Mlep.

We postulated that some proteins might be conserved in numerous *Actinobacteria*, represented in multiple datasets, and sought to identify Mtb orthologous proteins previously listed across multiple datasets. Our investigation uncovered the presence of 168 proteins that were conserved within two or three out of three datasets (Fig. 2A). We identified 70 proteins strictly conserved (SC) across the three datasets and 98 proteins found at least in two datasets. These 98 proteins were referred to as highly conserved proteins (HC) (Fig. 2A) and included 27 proteins conserved between Rjos‐Rop datasets, 40 conserved between Rjos‐Msmeg datasets, and the remaining 31 were conserved between Rop‐Msmeg datasets (Fig. 2A). Analysis of gene essentiality in Mtb showed again a homogenous distribution with 71 essential genes, 78 nonessential genes, 18 genes involved in growth advantage/defect, and 1 gene classified as uncertain, according to Dejesus and colleagues [44].

The distribution and classification of the conserved 168 orthologous proteins in Mtb FC is as follows: ‘*Cell wall and cell processes*’ (18/168–10.7%), ‘*Conserved*’ ‘*Hypotheticals*’ (23/168–13.7%), ‘*Information pathways*’ (33/168–19.6%), ‘*Intermediary metabolism and respiration*’ (54/168–32.1%), ‘*Lipid metabolism*’ (28/168–16.7%), ‘*Regulatory proteins*’ (6/168–9.5%), and ‘*Virulence, detoxification, adaptation*’ (6/168–9.5%) (Fig. 2B). Specific analysis of Mtb FC for SC and HC proteins across the datasets is also displayed (Fig. 2B).

Next, we investigated whether the 168 candidate proteins were conserved in other mycobacterial species, positing that they may be shared with other mycobacterial species. Thus, we looked for orthologs in both nonpathogenic and pathogenic species known to produce ILI, such as Msmeg, Mabs, Mmar, Mlep, and Mul (Fig. 2C). Our results show that 168/168 (100%) of the proteins are conserved in Msmeg and Mmar, 167/168 (99.4%) in Mabs, 164/168 (97.6%) in Mul, and 148/168 (88.1%) in Mlep, the etiologic agent of leprosy (Fig. 2C). All these information are combined in Table S4.

### Description of proteins involved in lipid metabolism at the ILI surface

Surprisingly, only 28 proteins were classified in the ‘*Lipid metabolism*’ FC, therefore representing only 16.7% (28/168) of the total proteins associated with ILI and approximately 12.0% (28/233) of the total proteins that have been originally classified in this FC by Cole and colleagues [51, 52, 53]. These results are consistent with the vision that recently emerged regarding the physiological role of ILI, which are structures primarily dedicated to lipid storage but also ensuring a wide range of functions to maintain cellular homeostasis in stringent conditions [54, 55].

Analysis of these 28 proteins (Table 1) showed that 20 are directly involved in phospholipids, fatty acids or mycolic acids biosynthesis, modification, or degradation processes (Fig. 2C).

**Table 1:** List of the 28 proteins belonging to the lipid metabolism FC identified as part of Mtb ILIome.

Dataset	Mtb ID	Gene name	Putative function	Essentiality	Msmeg ortholog	Mabs ortholog	Mmar ortholog	Mul ortholog	Mlep ortholog
Rop‐Rjos‐Msmeg	Rv0242c	*fabG4*	3‐ketoacyl‐ACP reductase	Nonessential	MSMEG_0372	MAB_4443	MMAR_0503	MUL_1166	ML2565
Rop‐Rjos‐Msmeg	Rv0243	*fadA2*	Acetyl‐CoA acyltransferase	Nonessential	MSMEG_0373	MAB_4442c	MMAR_0504	MUL_1167	ML2564
Rop‐Rjos‐Msmeg	Rv0270	*fadD2*	Fatty‐acid‐CoA ligase	Nonessential	MSMEG_0599	MAB_4340c	MMAR_0528	MUL_1191	ML2546
Rop‐Rjos‐Msmeg	Rv1206	*fadD6*	Fatty‐acid‐CoA ligase	Nonessential	MSMEG_5086	MAB_1342	MMAR_4232	MUL_0957	ML1346
Rop‐Rjos‐Msmeg	Rv1483	*fabG1*	3‐ketoacyl‐ACP reductase	Essential	MSMEG_3150	MAB_2723c	MMAR_2289	MUL_1491	ML1807c
Rop‐Rjos‐Msmeg	Rv1484	*inhA*	NADH‐dependent enoyl‐ACP reductase	Essential	MSMEG_3151	MAB_2722c	MMAR_2290	MUL_1492	ML1806c
Rop‐Rjos‐Msmeg	Rv1683	*–*	Long‐chain acyl‐CoA synthase and lipase	Nonessential	MSMEG_3767	MAB_2348	MMAR_2478	MUL_1660	ML1346
Rop‐Rjos‐Msmeg	Rv2187	*fadD15*	Long‐chain‐fatty‐acid‐CoA ligase	Nonessential	MSMEG_4254	MAB_1978c	MMAR_3231	MUL_3542	ML0887
Rop‐Rjos‐Msmeg	Rv3720	*–*	FAS	Nonessential	MSMEG_6284	MAB_0310c	MMAR_5236	*–*	ML2334
Rop‐Rjos‐Msmeg	Rv3791	*dprE2*	Decaprenylphosphoryl‐keto erythro pentose reductase	Essential	MSMEG_6385	MAB_0191c	MMAR_5353	MUL_4970	ML0108c
Rop‐Msmeg	Rv0468	*fadB2*	3‐hydroxybutyryl‐CoA dehydrogenase	Nonessential	MSMEG_0912	MAB_4094c	MMAR_0793	MUL_4537	ML2461c
Rop‐Msmeg	Rv2244	*acpM*	Meromycolate extension acyl carrier protein	Essential	MSMEG_4326	MAB_1878c	MMAR_3337	MUL_1305	ML1654
Rop‐Msmeg	Rv3800c	*pks13*	Polyketide synthase	Essential	MSMEG_6392	MAB_0180	MMAR_5364	MUL_4983	ML0101
Rop‐Msmeg	Rv2524c	*fas*	FAS	Essential	MSMEG_4757	MAB_1512	MMAR_3962	MUL_3818	ML1191
Rop‐Msmeg	Rv3790	*dprE1*	Decaprenylphosphoryl‐beta‐d‐ribose 2′‐oxidase	Essential	MSMEG_6382	MAB_0192c	MMAR_5352	MUL_4969	ML0109c
Rop‐Msmeg	Rv2483c	*plsC*	Transmembrane phospholipid biosynthesis bifunctional enzyme	Nonessential	MSMEG_4704	MAB_2455c	MMAR_3834	MUL_3764	ML1245
Rjos‐Msmeg	Rv2484c	*–*	Triacylglycerol synthase	Nonessential	MSMEG_4705	MAB_4544c	MMAR_3835	MUL_3765	ML1244
Rjos‐Msmeg	Rv1544	*–*	Ketoacyl reductase	Nonessential	MSMEG_0737	MAB_1537c	MMAR_2367	MUL_1543	ML0429c
Rjos‐Msmeg	Rv0437c	*psd*	Phosphatidylserine decarboxylase	Nonessential	MSMEG_0861	MAB_0639c	MMAR_0754	MUL_1386	ML0311c
Rjos‐Msmeg	Rv3229c	*desA3*	Linoleoyl‐CoA desaturase	Nonessential	MSMEG_1743	MAB_2148	MMAR_1315	MUL_2565	*–*
Rjos‐Msmeg	Rv0154c	*fadE2*	Acyl‐CoA dehydrogenase	Nonessential	MSMEG_0102	MAB_0255	MMAR_0374	MUL_4790	ML0737
Rop‐Rjos	Rv0895	*–*	Triacylglycerol synthase	Nonessential	MSMEG_6322	MAB_4544c	MMAR_5271	MUL_2057	ML1244
Rop‐Rjos	Rv3391	*acrA1*	Multi‐functional enzyme with acyl‐CoA‐reductase activity	Nonessential	MSMEG_1623	MAB_3710	MMAR_1153	MUL_0918	ML0862
Rop‐Rjos	Rv0400c	*fadE7*	Acyl‐CoA dehydrogenase	Nonessential	MSMEG_2466	MAB_0822	MMAR_0698	MUL_2825	ML0737
Rop‐Rjos	Rv2501c	*accA1*	Acetyl−/propionyl‐coenzyme A carboxylase alpha chain	Nonessential	MSMEG_4716	MAB_4539c	MMAR_3848	MUL_3779	ML0726c
Rop‐Rjos	Rv0824c	*desA1*	Acyl‐ACP desaturase	Essential	MSMEG_5773	MAB_0754c	MMAR_4856	MUL_0445	ML2185
Rop‐Rjos	Rv3130c	*tgs1*	Triacylglycerol synthase	Nonessential	MSMEG_5242	MAB_3551c	MMAR_1519	MUL_2420	ML1244
Rop‐Rjos	Rv2247	*accD6*	Acetyl/propionyl‐CoA carboxylase	Growth defect	MSMEG_4329	MAB_1876c	MMAR_3340	MUL_1302	ML1657

The first 16 include Rv0242c (FabG4), Rv0243 (FadA2), Rv0270 (FadD2), Rv1206 (FadD6), Rv1483 (FabG1), Rv2187 (FadD15), Rv0468 (FadB2), Rv3229c (DesA3), Rv0154c (FadE2), Rv2244 (AcpM), Rv3800c (Pks13), Rv2524c (Fas), Rv0400c (FadE7), Rv0824c (DesA1), Rv2501c (AccA1), and Rv2247 (AccD6).

**Fig. 3:**
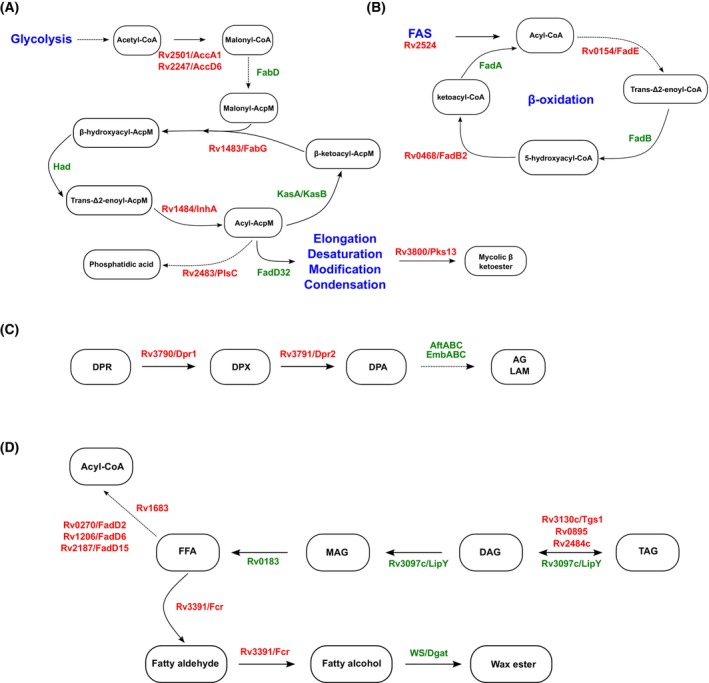
Schematic representation of the major pathways containing ILI‐associated proteins that belong in the ‘*Lipid Metabolism*’ FC. Schematic representation of (A) the biosynthesis of phosphatidic acid and mycolic acids from glycolysis‐derived acetyl‐CoA molecules; (B) FAS and β‐oxidation steps that are achieved through the action of identified ILI‐associated proteins; (C) ILI‐associated proteins Dpr1 and Dpr2‐mediated reactions involved in arabinogalactan and lipoarabinomannan biosynthesis. (D) FFAs, glycerolipids, and wax ester metabolism mediated by ILI‐associated proteins. All the proteins identified as ILI‐associated are highlighted in red in the scheme, whereas other proteins are displayed in green. FAS system, decaprenylphosphoryl ribose (DPR), decaprenylphosphoryl‐X (DPX), decaprenylphosphoryl‐d‐Araf (DPA), arabinogalactan (AG), lipoarabinomannan (LAM), FFA, monoacylglycerol (MAG), diacylglycerol (DAG), TAG, and wax ester synthase/diacylglycerol acyltransferase (WS/Dgat).

Three additional proteins, Rv1544, Rv3720, and Rv0437c, annotated as putative ketoacyl reductase, fatty‐acyl‐phospholipid synthase, and phosphatidylserine decarboxylase, respectively, were also identified. A simplified representation of the potential implication of these enzymes in Mtb lipid metabolism is shown in Fig. 3A,B.

Of note, our analysis unraveled the mycobacterial enoyl‐reductase enzyme Rv1484 (InhA), target of the anti‐TB drugs isoniazid and ethionamide, as potential actor at the surface of ILI. With KasAB, MabA, and HadABC, InhA forms the type II fatty acid synthase (FAS‐II), which elongates short‐chain fatty acids to long‐chain meromycolic acids (Fig. 3A). These latter are the biosynthetic precursors of mycolic acids, which are indispensable lipids for mycobacterial growth and survival [56, 57, 58].

Although the precise molecular component(s) responsible for acid‐fast staining positivity is still unknown, the common feature among all of the proposed mechanisms is the existence of an atypical lipid‐rich hydrophobic barrier that can be penetrated by phenol‐based stains but is impermeable to acido‐alcoholic solutions used in Ziehl‐Neelsen staining [59]. Seminal work on isoniazid showed that this drug inhibits mycolic biosynthesis, resulting in the loss of acid fastness in Mtb, highlighting the critical role of these lipid components in acid‐fastness [59, 60, 61]. Intriguingly, several studies reported that mycobacteria harboring Nile‐Red positive ILI tend to lose acid‐fastness and become more tolerant to front‐line drugs, including isoniazid [25, 55, 62]. When similar experiments were conducted in a Mtb *tgs1* deletion mutant, the bacilli failed to accumulate ILI *in vitro*, very few Nile‐Red positive bacteria were detected while most remained acid‐fast positive [55]. Based on these observations, it is tempting to speculate that the formation of ILI in nonreplicating bacteria is associated with alterations of the mycobacterial cell wall, which comprises the mycolic acid‐containing outer membrane. In this context, the localization of InhA and its enzymatic activity at the surface of ILI could be directly linked to these global changes.

Two additional major enzymes involved in cell wall biosynthesis were identified, the decaprenylphosphoryl‐β‐d‐ribose‐2′‐epimerase Rv3790 (DprE1) and the decaprenylphosphoryl‐2‐keto‐β‐d‐erythro‐pentose reductase Rv3791 (DprE2) (Fig. 3C). These two enzymes catalyze the formation of decaprenyl‐phospho‐arabinose, an essential precursor required for the synthesis of the arabinan moiety of arabinogalactan (AG) and lipoarabinomannan (LAM) (Fig. 3C). DprE enzymes are the therapeutic targets of new TB drug candidates: the benzothiazinones (BTZs) and the dinitrobenzamide derivatives (DNBs) [63, 64]. However, whether this specific localization impacts on AG/LAM synthesis or Mtb susceptibility to these drugs in ILI‐rich conditions remains to be investigated.

Triacylglycerol biosynthesis is subdivided into three major metabolic steps [4, 7]: (i) the import or *de novo* biosynthesis of acyl‐CoA molecules; (ii) the formation of various glycerol‐derived intermediates, and finally (iii) the sequential esterification of acyl‐CoA molecules onto these glycerol‐derived intermediates. Therefore, in this metabolic model, glycerol phospholipid metabolism plays a central role in the final steps to produce TAG [4, 7]. In addition to these 22 initial proteins, six additional proteins associated with fatty acids and glycerophospholipids metabolism were identified in this FC (Fig. 3D). This includes the Rv2483c and Rv2484c proteins, previously reported to participate in the conversion of lysophosphatidic acid into phosphatidic acid and TAG biosynthesis, respectively (Fig. 3D) [51, 65]. Indeed, the two corresponding genes are located next to genes encoding a putative carboxylesterase LipQ (Rv2485c), a probable glycerol‐3‐phosphate acyltransferase (Rv2482c), and a probable enoyl‐CoA hydratase (Rv2486), suggesting that they possibly be involved in TAG synthesis via the Kennedy pathway [65].

Two additional Tgs enzymes were identified in our analysis, the Rv0895 and the well‐characterized Rv3130c (Tgs1), consisting of the major TAG synthase in Mtb (Fig. 3D) [65]. The Mtb H37Rv genome possesses 15 *tgs*‐like genes, identified on their homology with the bifunctional wax synthase‐*tgs* gene of *A. calcoaceticus* [65]. Among these 15 proteins, Tgs1 has been identified as the final and primary enzyme involved in TAG synthesis *in vitro*, since deletion of the *tgs1* was associated with a severe defect in TAG production and reduced Nile‐Red positivity in under hypoxic conditions as well as in granuloma‐like models [55, 62, 65, 66].

Since ILI‐positive Mtb have been described in sputum from patients with active TB [13, 26], it is now commonly accepted that these organelles play a key role in the adaptation to pathophysiological environments such as the one encountered within granulomatous lesions. Indeed, upon stringent conditions, Mtb realigns its metabolism to produce TAG, which seem to be required for adaptation and overcoming multiple stresses [7, 18, 25, 55, 62]. One of the most characterized pathways involved in such adaptation is the upregulation of the dormancy survival regulon (Dos), which is coordinated by its two sensor histidine kinases, DosS and DosT, and its response regulator DosR [67]. This regulon comprises 48 genes (including *tgs1*) involved in lipid metabolism and anaerobic respiration [65, 68]. Among Mtb lineages, the Beijing lineage strains (L2 strains) show a constitutive overexpression of *dosR* compared with the non‐Beijing strains [69], where *tgs1* is always upregulated. As a result, *tgs1* is overexpressed by approximately 10‐fold, which leads to greater TAG accumulation. In addition, clinical isolates from this lineage also upregulate *tgs2*, independently of Dos, which has also been reported to increase TAG levels [70]. Such lipid‐rich phenotype has been proposed to be one of the phenotypic traits explaining the hypervirulence among these strains and their epidemiological effects [69, 70].

More recently, Mtb *tgs1* orthologs were identified in other mycobacterial species, including the emerging opportunistic pathogen Mabs. Among the seven *tgs* genes present within Mabs genome, one enzyme annotated *MAB_3551* was identified as the closest homolog to Tgs1 sharing 40% of sequence identity [33]. Molecular characterization of MAB_3551c indicated that this protein was essential for TAG production and the formation of ILI *in vitro* as well as in the foamy macrophage infection model [33].

One can hypothesize that the spatial distribution of Tgs enzymes within bacterial cells is bimodal. Indeed, time‐dependent analysis of TAG‐rich *Rhodococcus* cells by sophisticated electron microscopy approaches combined with immunolabelling experiments allowed us to establish that Dgat/Tgs enzymes were mainly localized at the plasma membrane level within specific microdomains [17]. This was recently confirmed in Mabs where the 7 Tgs were localized within the membrane fraction by cell‐fractionation and immunoblotting [33]. In contrast, Tgs1 has also been identified on the surface of mature ILI in *Mycobacterium bovis* BCG [31], suggesting that this critical enzyme might be either continuously associated with membrane‐derived premature ILI and remains associated upon releasing of cytosolic mature ILI, or displays a dynamic spatial distribution within the cells that fluctuates between these sub‐bacterial compartments.

Our analysis suggests also the presence of the acyl‐CoA reductase Rv3391 (*fcr1* or *acrA1*) [71], which is involved in the generation of fatty alcohol from acyl‐CoA to generate wax ester molecules (Fig. 3D). In Mtb, wax esters molecules have been observed to be involved in response to iron starvation [72], and described for being required to undergo into a nonreplicating persistent state when subjected to *in vitro* dormancy‐inducing conditions [71]. However, their direct contribution to Mtb pathogenesis remains unknown.

Finally, our analysis identified as well the conservation of the Rv1683 protein, a putative bifunctional long‐chain acyl‐CoA synthase/lipase (Fig. 3D), reported as an important regulator of TAG levels in *M. bovis* BCG [31]. Indeed, the Rv1683 and the BCG1721 protein are 100% identical, with the N‐terminal domain that is thought to express acyl‐CoA synthase/ligase activity whereas the putative C‐terminal harbors a lipase domain, typified by the consensus GXSXG motif and is homologous to the catalytic domain of the human gastric lipase [31]. Overexpression of *M. bovis* BCG *BCG1721* gene had a dual impact on TAG levels and ILI formation/consumption processes when assessed under nonreplicating or resuscitating conditions in the Wayne model [31]. The authors demonstrate that long‐chain TAG levels significantly increased under the nonreplicating states when the *BCG1721* gene was overexpressed [31]. However, overexpression of *BCG1721* during the reactivation phase was associated with an increase in TAG lipolysis; this process was impaired when the inactive *BCG1721* ^*S150A*^ gene in which the catalytic serine replaced by an alanine residue in the lipase domain was overexpressed [31]. Thus, many questions regarding the physiological function of this bifunctional enzyme remain to be discovered.

Importantly, these 28 proteins were also found in other mycobacterial species with the exception of Rv3720 and Rv3229 orthologs in Mul and in Mlep, respectively (Table 1). This suggests that the few enzymes that belong to the ‘*Lipid metabolism*’ FC are likely to play a key role in the formation, maintenance, and degradation of ILI in various mycobacteria.

Since the presence of ILI is associated with a nonreplicating persistent‐like phenotype, antibiotic tolerance, and some hypervirulent features, targeting ILI metabolism may be viewed as a potent antivirulence strategy and/or a potential therapeutic option in the context of mycobacterial‐related diseases. Therefore, dissecting the fundamental contribution of each of these proteins at the molecular and cellular level should be considered as a top priority.

### Identification of structural motifs and molecular binding features of ILI‐associated proteins—Amphipathic helices and the case of Tgs1

Understanding how ILI‐associated proteins bind to the lipid surface of these organelles represents a real challenge. However, pioneering studies have proposed several mechanisms by which these proteins interact with ILI.

Since the phospholipid monolayer constitutes the main biological interface, LD‐ or ILI‐associated proteins must display well‐defined physico‐chemical and/or structural properties to interact with such surface. Indeed, electrostatic interactions, hydrophobic binding regions, β‐hairpins, and amphipathic helices are, until today, the known patterns for LD‐associated protein localization [73]. While β‐hairpins have been extensively studied for protein targeting on LD [74], little is known about their role in prokaryotes, especially in mycobacteria. However, it was proposed that ILI‐associated proteins found in mycobacteria heavily rely on amphipathic helices for ILI binding [32]. The presence of multiple amphipathic patterns using the Heliquest Prediction algorithm suggests that this structural motif may govern protein targeting to ILI in Msmeg [32]. This prompted us to further test this model in our dataset according to their experimental strategy and search for amphipathic motifs that could be conserved in the 168 HC ILI‐associated candidate proteins. Through our analysis, we identify that approximately 56% (95/168) of the candidate proteins displayed a putative amphipathic helix. Information about each individual protein is available in the Table S4. Interestingly, these results are in accordance with previously published observations, suggesting that the presence of such motif could be important but not essential for targeting proteins to the ILI surface [32].

Among the 28 ‘*Lipid metabolism*’ FC proteins identified as potential core components of Mtb ILIome, 20 show putative amphipathic helices, possibly constituting strong binding motifs. Of note, Tgs1, the major TAG synthase in multiple mycobacterial species, possesses such a motif [31, 33, 35, 75, 76].

**Fig. 4:**
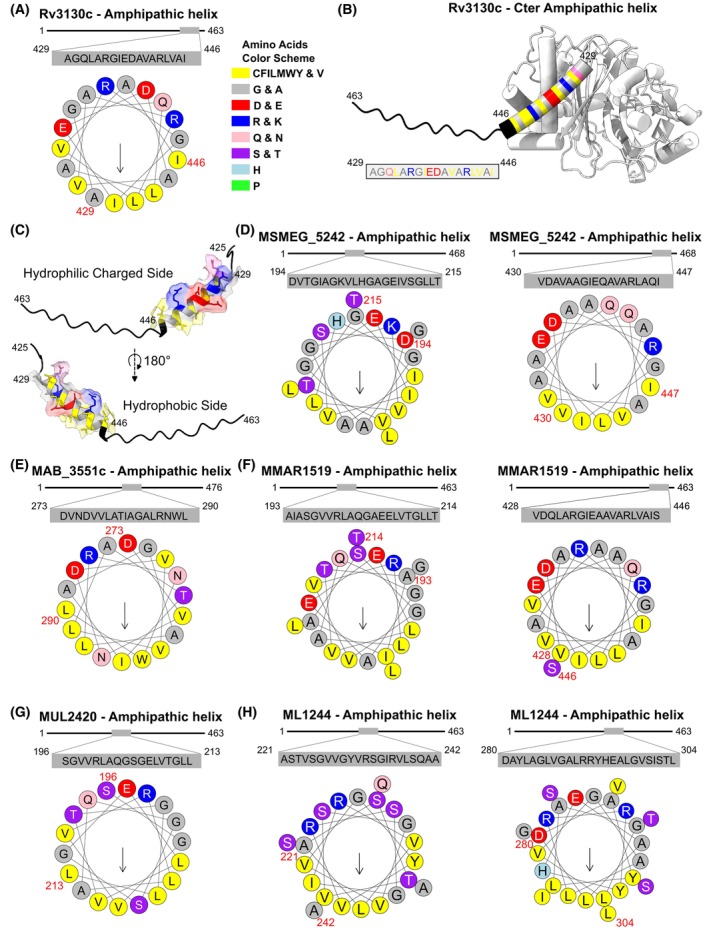
Amphipathic helices from Tgs proteins as putative ILI‐binding motifs. (A) Representation of Mtb Tgs1 C‐terminal amphipathic helix as a helical wheel diagram. The helix comprises residues 429 to 446/463 of the protein. The arrow indicates the angle of the mean hydrophobic moment toward the hydrophobic face of the amphipathic helix. The amino acids color code is summarized on the right part of the panel. (B) Overall view of the AlphaFold2 prediction of Mtb Tgs1 3D structural model. The protein is displayed in white with the extreme C‐terminal end that is highlighted in black and the amphipathic helix (429–446) is shown as multi‐colored cylinder according to the amino acids color code displayed in (A). (C) A zoomed version of the C‐terminal end of Mtb Tgs1 and the amphipathic motif is displayed from two distinct views, highlighting the hydrophilic/hydrophobic sides of the helix. (D–H) Representation of Tgs1 putative amphipathic helices as helical wheel diagrams from multiple mycobacterial strains. The arrow indicates the angle of the mean hydrophobic moment toward the hydrophobic face of the amphipathic helix. The amino acids color code is summarized on the right part of the panel (A).

Since previous reports have proposed that Tgs proteins, and more particularly Tgs1, could localize and interact with the ILI surface through these conserved binding motifs involving amphipathic helices [32], we investigated whether we could identify putative ILI‐binding motifs contained within the mycobacterial Tgs1 proteins. We first started with the most characterized Tgs, and confirmed the presence of a putative amphipathic helix motif in the Tgs1 of Mtb by using Heliquest‐based predictions combined with structural analysis. Results predicted an alpha‐helix fold in the C‐terminal region that displays putative amphipathic properties (Fig. 4A). Structural investigations using AlphaFold2 predictions confirmed that these 18 consecutive residues, formed an amphipathic patch located within an alpha‐helix at the C terminus position 429–446/463 of Tgs1, which agrees with previous observations (Fig. 4B,C) [32]. Interestingly, according to the AlphaFold2 model, this C‐terminal helix is predicted to be surface exposed, therefore fully accessible to bind the lipid interface without major structural rearrangements. Analysis of this C‐terminal patch revealed two interfaces with well‐defined biochemical properties (Fig. 4C). The first one is essentially composed of hydrophilic charged residues (ERDQ residues), which might facilitate the interaction of the protein with negatively charged phospholipid heads, other proteins, or the cytosolic environment. On the contrary, the second side is mainly composed by hydrophobic amino acids (AVIL residues) that form the putative ILI‐binding motif (Fig. 4C).

Overall, this analysis suggests that these two sides may constitute a strong binding motif that facilitates the insertion of the helix into the phospholipid monolayer, enabling the enzyme's activity onto the ILI surface.

We next investigated the conservation of the amphipathic helix in other mycobacterial Tgs1 proteins. By combining PSIPred and Heliquest analysis, we identified one or two amphipathic helices, ranging from 14 to 25 residues, as putative lipid‐binding sites for each of the tested Tgs1. Results from Msmeg (Fig. 4D), Mmar (Fig. 4F), and Mlep (Fig. 4H) showed that two putative motifs were detectable, with one motif that was very similar to the one identified at the C‐terminal of Mtb Tgs1. Regarding Mabs and Mul, only one amphipathic motif was identified, which was not located at the C‐terminal, but rather in the middle of the protein sequence (positions 273‐290/476 and 196‐213/463 for Mabs and Mul, respectively) (Fig. 4E,G). Using AlphaFold2 predictions, we checked that all the identified amphipathic helices were surface exposed (data not shown) and not buried within the structure or the catalytic site of the proteins. All of them were surface‐exposed and, therefore, could be clearly involved in these interfacial interactions. Next, we investigated whether these candidates harbor strong hydrophobic binding regions or patches with high electrostatic potential [77, 78], as observed for peripheral proteins interacting with phospholipids membrane [79]. However, we could not detect these features, suggesting that the amphipathic motifs are likely to represent the primary motif responsible for ILI targeting.

### ILI‐associated proteins from other functional categories—A cornerstone for metabolic adaptation?

Herein, we have listed 168 proteins that might be associated with ILI in the tubercule bacilli but also in other mycobacterial species, constituting a potential ILIome core. Unexpectedly, only 28 proteins were classified into the ‘*Lipid metabolism*’ FC and the remaining 140 proteins (84%) belonged to 6 other FC: ‘*Information pathways*’, ‘*Cell wall and cell processes*’, ‘*Intermediary metabolism and respiration*’, ‘*Regulatory proteins*’, ‘*Conserved hypotheticals*’, and ‘*Virulence, detoxification and adaptation*’. These findings clearly support the concept that has recently emerged regarding the dynamic composition and multi‐faceted roles of ILI in the mycobacterial lifecycle where these structures are not just limited to lipid and energy storage [7].

Among LD‐associated proteins, the first identified proteins were the perilipins (PLINs), which were described as scaffolding proteins responsible for the LD structure integrity [80, 81, 82]. These proteins play a crucial role in LD formation, maintenance, and degradation, highlighting that some key actors that are not directly involved or referenced as ‘*Lipid metabolism*’ proteins may constitute a corner stone for TAG accumulation under the form of LD. Interestingly, structural proteins that might have a role similar to PLINs have been also identified in prokaryotes. The regulator protein TadA (named for ‘*triacylglycerol accumulation deficient*’) was identified as essential for TAG accumulation and ILI formation in *R. opacus* PD630 [83]. Studies showed that this protein belongs to the heparin‐binding family and contributes to regulating the size and shape of ILI [83]. The TadA ortholog HbhA was later identified in Msmeg [84, 85]. In *R. jostii* RHA1, Ding *et al*. identified another ILI‐associated protein PspA responsible for the regulation of ILI size and homeostasis [30]. A PspA ortholog was also found and recently characterized in mycobacteria where it localizes to the ILI surface, regulates their number and size, and impacts survival upon hypoxia‐induced dormancy [86]. Additionally, the PLIN‐like protein Rv1039c (PPE15 or MPER1) was identified in Mtb as required for optimal TAG accumulation and the display of key nonreplicating features within *in vitro* models of dormancy, including a three‐dimensional human granuloma model [87].

These observations highlight that some proteins which do not belong the ‘*Lipid metabolism*’ FC, can have very important role in the formation, maintenance, or degradation of ILI. This idea is also supported by the recent discovery of an unconventional DNA binding feature of these membrane‐less organelles. Indeed, it was established in different eukaryote organisms that LD binds to the nucleus, histones, or nucleic acids to help nuclear lipid homeostasis or even act as an antibacterial defense system [88, 89, 90, 91]. Likewise, Zhang and collaborators demonstrated that such peculiar feature was conserved in prokaryotes, and they observed that ILI from *R. jostii* binds to DNA to prevent genotoxic stress [92]. In addition, ILI has been proposed to serve as anchor which contributes to the detoxification process upon excessive lipid or ROS levels [93]. In multiple organisms, TAG production protects against FFA and reductive stress, therefore limiting lipotoxicity [18, 94, 95]. Thus, it is not surprising to find that most of these proteins belong to ‘*Virulence, detoxification and adaptation*’ and the ‘*Intermediary metabolism and respiration*’ FC.

In summary, our analysis revealed that numerous proteins from different FCs are shared among distinct TAG‐producing organisms that belong to the *Actinobacteria* phylum. These proteins are HC in Mtb, suggesting that a core of specific proteins might form a dedicated ILI‐associated proteome that localizes at the surface of this particular organelle. The presence of this ILIome core reinforces the idea that ILI are not just energy storage organelles, but are more complex structures with multiple physiological functions in prokaryotes, and specifically in the mycobacterial lifestyle. Moreover, our study uncovered the presence of amphipathic helix in numerous ILI‐associated proteins, prompting us and others to propose that such motif could be essential for binding and targeting ILI [32]. While these investigations and the proposed amphipathic patches‐mediated binding model by which these proteins interact with the ILI surface remain hypothetic, requiring further experimental validations [32].

Finally, since conventional ILI purification strategies rely on mechanical disruption of bacterial cells followed by ultracentrifugation separation, it is very likely that the isolated ILI‐associated proteomes harbor cross‐contaminating proteins. This raises questions about the accuracy of these subcellular localization studies. To overcome these limitations, the development of new technological modalities that allow to noninvasively investigate ILI‐associated proteome is urgently needed [8]. The recent emergence of proximity labelling technologies has the potential to circumvent these limitations and offer further insights into the exact composition of the ILIomes [31]. Furthermore, these approaches have not only the potential to prevail over some of the spatial limitations, but they should open new avenues regarding the temporal dynamics of ILI‐associated protein recruitment. In that context, we believe that such proximity labelling techniques should be implemented at different stages of organelle formation and consumption, to finely dissect the dynamics interactions that occur at the ILI surface.

We hope that this study will provide relevant information and concepts in order to further delineate and investigate the nature and function of ILI‐associated proteins in Mtb and other mycobacterial species, and will contribute to a better understanding of the cellular and molecular mechanisms underlying ILI biology.

## Conflict of interest

The authors declare no conflict of interest.

### Peer review

The peer review history for this article is available at https://www.webofscience.com/api/gateway/wos/peer‐review/10.1002/2211‐5463.13721.

## Authors contributions

PS proposed conceived and led the study. SC secured funding and co‐advised this work with LK. TD and IM performed most of the *in silico* analysis with the guidance of PS and SC. PS and TD edited the figures. All authors provided intellectual input by organizing, analyzing, and/or discussing data. PS wrote the manuscript with input from TD, LK, and SC. All authors read the manuscript and provided critical feedback before its submission.

## Supporting information


**Table S1.** Computational identification of Mtb orthologous ILI‐associated proteins from Rjos RHA1.

**Table S2.** Computational identification of Mtb orthologous ILI‐associated proteins from Rop PD630.

**Table S3.** Computational identification of Mtb orthologous ILI‐associated proteins from Msmeg mc^2^ 155.

**Table S4.** List of the 168 Mtb orthologous ILI‐associated proteins that are shared in at least two or three datasets analyzed and form Mtb ILIome.

## Data Availability

Any additional data that support the findings of this study are available upon reasonable request from the corresponding authors at canaan@imm.cnrs.fr or psantucci@imm.cnrs.fr.
